# The Interactive Effect of Tonic Pain and Motor Learning on Corticospinal Excitability

**DOI:** 10.3390/brainsci9030063

**Published:** 2019-03-16

**Authors:** Erin Dancey, Paul Yielder, Bernadette Murphy

**Affiliations:** University of Ontario Institute of Technology, Faculty of Health Sciences, Oshawa, ON L1G 0C5, Canada; erin.dancey@uoit.ca (E.D.); Paul.Yielder@uoit.ca (P.Y.)

**Keywords:** transcranial magnetic stimulation (TMS), input–output (IO) curves, motor learning, acute pain, sensorimotor integration

## Abstract

Prior work showed differential alterations in early somatosensory evoked potentials (SEPs) and improved motor learning while in acute tonic pain. The aim of the current study was to determine the interactive effect of acute tonic pain and early motor learning on corticospinal excitability as measured by transcranial magnetic stimulation (TMS). Two groups of twelve participants (*n* = 24) were randomly assigned to a control (inert lotion) or capsaicin (capsaicin cream) group. TMS input–output (IO) curves were performed at baseline, post-application, and following motor learning acquisition. Following the application of the creams, participants in both groups completed a motor tracing task (pre-test and an acquisition test) followed by a retention test (completed without capsaicin) within 24–48 h. Following an acquisition phase, there was a significant increase in the slope of the TMS IO curves for the control group (*p* < 0.05), and no significant change for the capsaicin group (*p* = 0.57). Both groups improved in accuracy following an acquisition phase (*p* < 0.001). The capsaicin group outperformed the control group at pre-test (*p* < 0.005), following an acquisition phase (*p* < 0.005), and following a retention test (*p* < 0.005). When data was normalized to the pre-test values, the learning effects were similar for both groups post-acquisition and at retention (*p* < 0.005), with no interactive effect of group. The acute tonic pain in this study was shown to negate the increase in IO slope observed for the control group despite the fact that motor performance improved similarly to the control group following acquisition and retention. This study highlights the need to better understand the implications of neural changes accompanying early motor learning, particularly while in pain.

## 1. Introduction

There is evidence that pain negatively impacts the neuroplasticity associated with motor output [1,2] and negates the increases in somatosensory evoked potential (SEP) peaks that would otherwise occur following early motor learning [3,4]. Motor learning is the acquisition of new muscle patterns in order to improve motor performance [5]. Motor learning induces neuroplasticity in the motor cortex (M1): An expansion of motor areas [6,7,8,9] and changes in the kinematics of movements and facilitation of the motor evoked potentials (MEPs) [10,11]. The interactions between pain and motor learning are complicated and few experiments have examined the interactive effect of acute tonic pain and early motor learning on corticospinal excitability (CSE) in healthy humans. In addition, while there is some evidence that pain during motor learning interferes with acquisition [12,13,14], other work has shown no impact of pain on acquisition [15,16,17] and improved acquisition in the presence of acute pain [3,4]. Previous work using a tonic cutaneous pain model demonstrated improved early motor learning with remote acute pain (applied to the same limb) as compared to a control group [3,4] and with local pain as compared to remote pain [4]. Therefore, as the neuroplasticity associated with early motor learning is mediated by alterations in attention [18,19,20,21], these differing outcomes may be attributed to the location (local versus remote) as local pain may help to focus attention on the effector performing the task leading to enhanced performance. In addition, as manipulating arousal through pharmacological means has been shown to improve performance [22], the increase in arousal associated with acute tonic may have a positive impact on motor performance. Another factor may be the type of nociceptive input (muscle versus cutaneous). Experimental muscle pain (induced by the injection of hypertonic saline) tends to increase with movement in certain directions, leading to altered motor control strategies in an effort to avoid the pain [1]. In contrast, cutaneous pain does not increase with movement and therefore does not interfere with motor learning. An acute tonic pain model (capsaicin cream) that does not lead to increased pain with movement was chosen for this study in order to elucidate some of the differences between the type of pain (cutaneous versus muscular) and early motor learning outcomes. The topical application of capsaicin cream is a widely used experimental pain model [23,24,25] that elicits activation in C-nociceptors inducing central sensitization and a region of hyperalgesia [26,27].

Transcranial magnetic stimulation (TMS) has been utilized to study alterations in CSE that occurs with acute and chronic pain as well as following motor learning. TMS studies of patients in chronic pain generally demonstrate decreased M1 excitability [28,29] although increased M1 excitability has been found in patients suffering from phantom limb pain [29]. In terms of acute pain, research demonstrates that experimental muscle pain modulates neuromuscular control through differential activation of muscle groups [30,31,32,33]. Although there are divergent effects of acute pain on M1 excitability as it increases excitability under certain circumstances [34], the preponderance of the literature demonstrates that there is generally a reduction in excitability [35,36,37]. Acute pain leads to alterations in excitability for differing muscles, and these changes in excitability seem to be dependent on which muscles need to be activated or inhibited to produce a protective motor strategy [1]. While acute pain generally reduces excitability, the alterations in M1 excitability following improved motor performance vary considerably across different studies. This may depend on the number of trials during motor learning as there is increased excitability of the M1 in the early stage of motor learning [38,39]. The early learning stage occurs in which there is a within-session improvement induced by a few trials on a time scale of minutes [40]. Following this early (fast) learning, there is slow increase in performance gains, and this is referred to as slow learning [40]. This phase in motor learning is a result of the consolidation of experience dependent changes in the cortex, triggered by learning. There is also a body of literature that has examined the interactive effect of pain and motor learning on somatosensory evoked potentials (SEPs) which can be used to assess the areas of the brain that are involved in sensorimotor integration [3,4,41,42]. Following local, remote, and contralateral cutaneous pain, there are decreases in an early SEP peak (P25) and differential changes in cortical SEP peaks for a control group following motor learning acquisition [42]. These findings were not observed for the capsaicin group, suggesting that acute pain may have negated alterations in SEP peaks that would have otherwise ensued following pain-free motor learning acquisition [42].

The literature demonstrates that there are conflicting findings in terms of the interactive effect of acute pain and motor learning on CSE. Previous studies have suggested that pain may interfere with learning-induced neuroplasticity [14]; however, other research findings demonstrated that pain improves motor learning [3] or has no effect when efforts were taken to ensure that the pattern of movement was not altered [16,43]. Research investigating the effect of local pain on motor learning found that following motor learning there were increases in MEPs for the control groups, with a lack of change in MEPs for the local acute pain groups [13,16]. It was hypothesized that local pain negatively impacts the CSE increases that occur with motor learning [13,16]. Ingham and Tucker [43] extended this research by examining how the location of the painful stimulus impacts CSE and found that there were no changes in MEPs for either group (control or hypertonic pain, both local or remote) following a finger adduction task. However, following motor learning, they did show that there was a reduction in TMS-evoked finger movement in the abduction direction for both the control and local pain condition, with no alterations following remote pain. This suggests that the location of pain may impact the CSE associated with motor learning. Furthermore, Mavromatis [44] examined the interactive effect of locally applied capsaicin cream and a motor task on corticospinal excitability. This study utilized short-interval intracortical inhibition (SICI) and found that the control group had an increase in excitability that was not observed for the capsaicin group but did not find a negative impact of local pain on motor learning outcomes. TMS input–output (IO) curves provide an opportunity to study CSE across a range of stimulus intensities [45]. When examining the IO curve, the slope of the linear component represents CSE [46] and therefore may provide a more robust way to explore the interactive effect of motor learning and pain on CSE than changes in MEP amplitude at a single stimulation intensity.

Our current study examines the effect of remote pain (capsaicin applied to the elbow) on the hand performing the task, and is therefore a distinct extension of previous research that has investigated the interactive effect of local pain and motor learning on CSE [14,17,44]. This paradigm allows us to examine the interactive effect of motor learning and acute pain on CSE. These findings on healthy individuals can then be contrasted with those suffering from chronic pain or other neurodegenerative disorders in order to understand the neurophysiological mechanisms at work. In addition, the application of capsaicin to the elbow is a model for lateral epicondylitis and thus the effect of elbow pain on motor performance is of relevance to ergonomics. This study provides an appealing model to investigate the interaction of pain and motor learning and it is also complimentary to our previous work, which found that remote cutaneous pain negated the somatosensory evoked potential (SEP) peaks that occurred in a control (non-capsaicin) group following motor learning [3,4].

A challenge with motor learning paradigms is that every repetition of the task, including repeated baseline testing, leads to a potential learning effect. Given that our primary interest was the effect of pain on motor learning, as distinct from the effect of pain itself on performance, and that our past work [41] has shown that pain improves performance, we opted to test baseline performance in the presence of pain. This enabled us to measure the effect of pain on motor learning as distinct from the impact of pain itself. In line with our previous work [4,41], we expected that the group which performed their novel motor acquisition task in the presence of acute tonic pain would show improved accuracy as compared to a control group.

Our primary hypothesis was that a motor acquisition task performed in a pain-free condition (controls) as compared to acute tonic pain (capsaicin group) would show an increase in the mean slope of the TMS IO curves. The results of this study will contribute to our understanding of how acute pain impacts CSE in response to novel motor acquisition, as well as provide insight on the relationship between excitability changes and the impact on motor skill retention when acquired during acute tonic pain.

## 2. Methods

### 2.1. Methods Overview

Two groups of twelve participants (6 males, 18 females; aged (M 20.2 1.31 SD)) were volunteers recruited from the student population at the University of Ontario Institute of Technology. Participants completed a confidential health history form to rule out any conditions which might impact normal somatosensation and/or motor function. This included recent cervicothoracic injury, chronic pain, neurologic conditions, and medication use. Participants self-reported their handedness in order to determine where the topical cream would be applied. This study was approved by the University of Ontario Institute of Technology Research Ethics Board and each participant gave both verbal and written informed consent. This study was performed according to the principles set out by the Declaration of Helsinki for the use of humans in experimental research.

**Figure 1:**
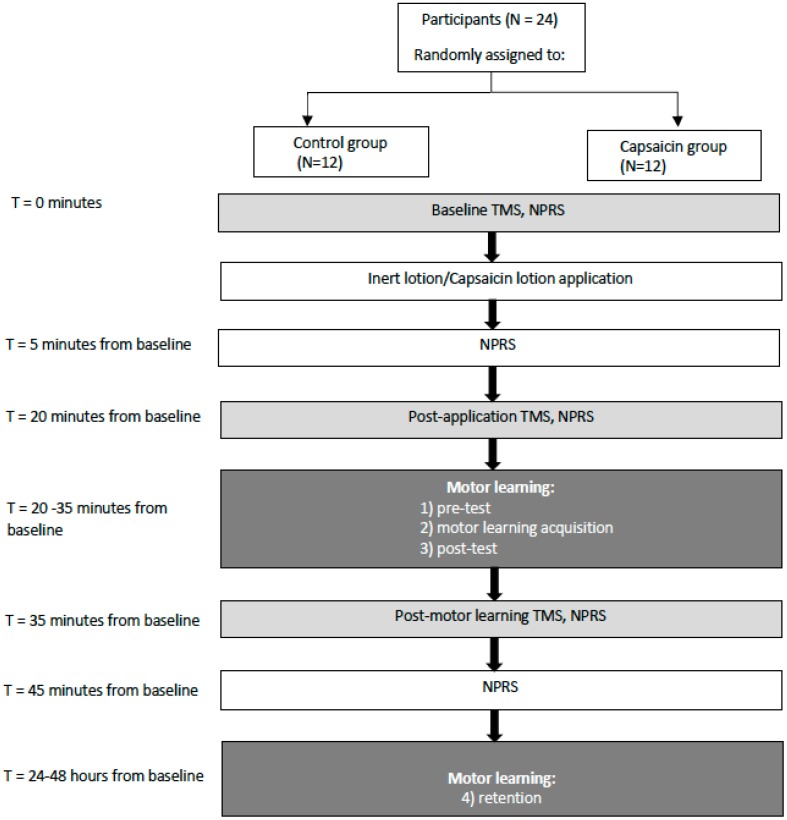
Schematic of the protocol illustrating that transcranial magnetic stimulation input–output (TMS IO) curves were performed at baseline, 20 min post-application, and then following the acquisition phase (45 min from baseline). Somatosensory evoked potentials (SEPs), numeric pain rating scale (NPRS).

The effect of acute tonic pain on CSE was assessed by performing TMS IO curves at baseline, at 20 min post-application, and then following the acquisition phase (45 min from baseline) (See Figure 1 for a schematic of the protocol). Participants in the capsaicin group received a topical application of capsaicin (0.075% Zostrix, New York, NY, USA) while the control group received a topical control skin lotion (Life Brand, Shopper’s Drug Mart, ON, Canada). The topical creams were applied to a 50 cm^2^ area on the lateral aspect of the dominant elbow.

### 2.2. Outcome Measures

The outcome measures for this study included pain (numeric pain rating scale (NPRS)), mean motor error on the motor task (%), and the slope of the IO curves.

#### 2.2.1. Pain

Pain was measured using an NPRS in which participants classified their pain intensity from 0–10 [47]. Participants in both groups rated their pain at baseline, post-application (5 min), post-application (20 min), following the acquisition phase (35 min), and following the last round of TMS measurements (45 min).

#### 2.2.2. Motor Learning Task

**Figure 2:**
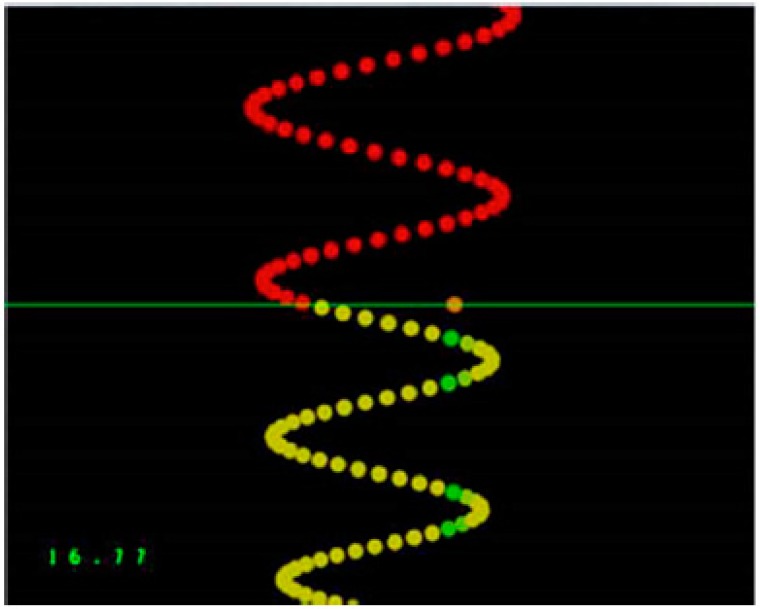
A photograph of the motor learning task that was performed by each participant. The traces consist of a series of dots which the participant tracked when the trace passed a horizontal line. The orange dot shows the location of the participant’s cursor along the horizontal line. Motor error was calculated as the average distance that the cursor was from the optimal trace.

A custom Leap Motion software tool (Leap Motion, Inc., San Francisco, CA, USA) was used to design the tracing task. Participants were asked to use their dominant thumb to trace sequences of sinusoidal waves with varying amplitude and frequency on an external touchpad (Logitech, Inc., Fremont, CA, USA). The trace moved vertically down a computer screen passing a horizontal line where the participants were attempting to follow the trace as accurately as possible using their dominant thumb. As described previously, participants were given visual feedback for each dot in the trace, with green indicating they were on the optimal trace and yellow indicating that they were further away [42]. There was a pre-test, an acquisition phase which took approximately 15 min, a post-acquisition test, and a retention test 24–48 h later. The pre-test, post-acquisition test, and retention tests all took 4 min to complete and consisted of four previously selected sinusoidal patterns of varying frequency and amplitude, as determined previously (See Figure 2) [48]. The traces consisted of a series of dots and each trial consisted of 500 dots. The acquisition phase consisted of a total of twelve traces as each version of the sinusoidal pattern was performed three times. The participants used the abductor pollicis brevis (APB) muscle as they were required to sweep their dominant thumb from right to left. As previously described [41], the software calculated the distance that the participant’s cursor dot was from the optimal trace by recording the mean distance the participant’s cursor was from each dot. Mean motor error expressed the percentage that the participant’s trace was from the original optimal trace, with larger numbers indicating more motor error and therefore reduced performance of the motor task.

#### 2.2.3. Transcranial Magnetic Stimulation

Focal TMS was performed over the hand region of the dominant M1 using a figure-eight coil (outer diameter 10 cm) linked to two Magstim 200 stimulators connected with a BiStim unit (Magstim Co., Whitland, Dyfed, UK). The coil was positioned rotated approximately 45 degrees away from the mid-sagittal line with the handle pointed backwards. This orientation induced the current perpendicular to the central sulcus, and therefore the TMS coil stimulated corticospinal neurons trans-synaptically [49,50]. The optimal coil position for inducing MEPs in the APB muscle was established as the site where stimulation at a suprathreshold level generated the largest MEPs (after averaging ten stimuli). The site was marked on a cap with a marker in order to confirm that the coil was correctly placed throughout the study. The location of the cap was checked throughout the experiment to ensure that it hadn’t moved. Resting motor threshold (rMT) was found utilizing the guidelines set forth by Rossini et al. [51], and thus we utilized the lowest stimulator intensity that in five out of ten sessions evoked a MEP of at least 0.05 mV, while the participant was at rest.

#### 2.2.4. Electromyography Recording

Surface electromyography (EMG) recordings were obtained from the APB muscle with surface Ag–AgCl electrodes. The electrodes were positioned over the APB, while the reference electrode was located over the lateral epicondyle of the same limb (there was no overlap between the application site of the inert lotion/capsaicin and the reference site). We requested that participants maintain a relaxed position throughout the study and monitored their level of muscle contraction using continuous visual EMG feedback. The recorded EMG signal was amplified, band-pass filtered (1000×), and digitized.

#### 2.2.5. IO Curves

The intensities used to develop the TMS IO curves were established for each participant using their rMT attained at the start of the experiment (prior to application of the capsaicin lotion/inert lotion) (See Table 1). As pain intensity changed throughout the experiment, it was important to collect recruitment curve data as efficiently as possible. Therefore, in keeping with previous research [46,48], we utilized magnetic stimuli between 90 and 140% of rMT (in 10% increments), as this range encompassed the linear portion of the curve for the majority of participants. Twelve stimuli were given at each stimulus intensity with the interstimulus interval set at 5 s. Therefore, a single IO curve session consisted of 72 stimuli.

### 2.3. Data Analysis

For the NPRS measurements, a repeated measures ANOVA with factors TIME (baseline, post-application (5 min), post-application (20 min), post-acquisition test (35 min), post-acquisition test (45 min)) and GROUP (control versus capsaicin) was performed.

Mauchly’s test of sphericity and the Shapiro–Wilk test for normality were run on the accuracy data (mean motor error). To compare accuracy between groups, a repeated measures ANOVA with factors TIME (pre-test versus post-acquisition test versus retention test) and GROUP (control versus capsaicin) was performed on the accuracy data with post-hoc ANOVA testing with a Bonferroni correction if there were significant main effects. Given that two past studies [41,42] showed that capsaicin leads to better motor performance, it was possible that there would be baseline performance differences between the two groups. Therefore, the accuracy data was also normalized to each individual’s pre-test accuracy data, and a repeated measures ANOVA with factors TIME (pre-test versus post-acquisition test versus retention) and GROUP (control versus capsaicin) was performed on the normalized data, in order to enable comparison of the relative improvements in performance between groups.

MEP amplitude measurements were taken peak-to-peak and averaged for each intensity. This file was exported to Microsoft Excel 2010 (version 16.5), and we took the average of the 12 stimuli for every intensity and graphed these results. The slope of the linear component of the IO curve was calculated and exported to IBM SPSS Statistics. R^2^ values were calculated to illustrate how well the slope fit the linear portion of the IO curve. The plateau phase was excluded for participants that had levelling off at the lower intensity (90% rMT), and thus only the slope of the curve from the 100% to 140% intensity was included in the final analysis for all of the participants. MEP amplitudes were normalized to baseline values to account for variability between participants at baseline and to allow for between participant comparisons. Mauchly’s test of sphericity and the Shapiro–Wilk test for normality were run on the slope data. IBM SPSS Statistics for Windows, Version 19.0 (IBM Corp., Armonk, NY, USA) was utilized for statistical analysis. Statistical significance was set at *p* < 0.05. To explore the interactive effect of acute tonic pain and the motor task on the IO slopes, a two-way repeated measures ANOVA with factors TIME (baseline, post-application, post-acquisition test) and GROUP (control versus capsaicin) was performed with post hoc ANOVA tests with a Bonferroni correction if a significant main effect was found.

## 3. Results

There was a total of 24 participants, with 12 participants in the capsaicin group (9 females, 3 males; aged 19–22 (M 19.9 SD 0.9)) and 12 participants in the control group (9 females, 3 males; aged 19–23 (M 20.7 SD 1.4)). Participants were stratified by sex to ensure equal numbers of males and females in each group.

### 3.1. Pain Ratings

**Figure 3:**
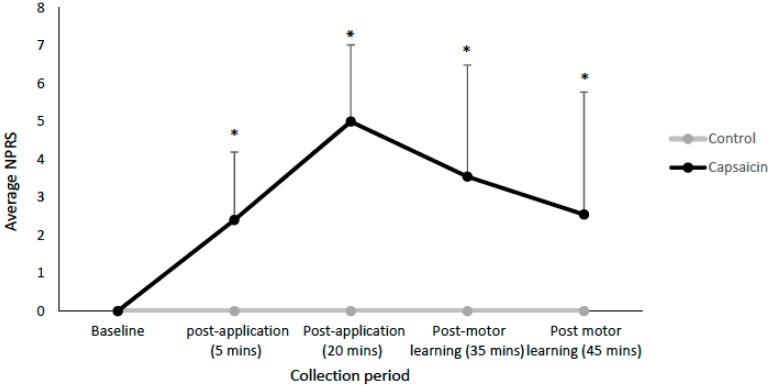
Line-graph depicting the average NPRS ratings for the capsaicin and control groups. Significant differences are indicated by asterisks (all *p* < 0.05). Error bars represent the standard deviation.

There were significant differences in subjective pain ratings as compared to baseline for the capsaicin group 5 min post-application (F(1,11) = 54.55, *p* < 0.001), 20 min post-application (F(1,11) = 286.00, *p* < 0.001), post-acquisition test (35 min mark) (F(1, 11) = 11.64, *p* < 0.01), and post-acquisition test (45 min mark) (F(1,11) = 7.05, *p* < 0.05). The average NPRS ratings are shown in Figure 3. The participants in the control group did not report any pain.

### 3.2. Behavioural Data

**Figure 4:**
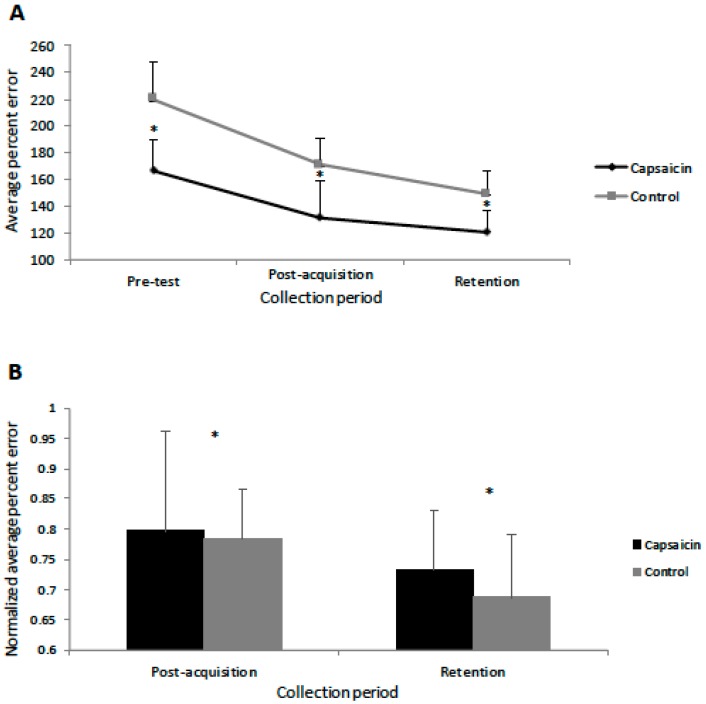
(**A**) Line-graph depicting the average percent motor error (in %) for the capsaicin and control groups. Significant differences between the groups are indicated by asterisks (all *p* < 0.005). Error bars represent the standard deviation. (**B**) Bar graph depicting the normalized percent error for the capsaicin and control groups. Significant differences (from pre-test) are indicated by asterisks (all *p* < 0.005) for both groups post-acquisition and at retention with no significant differences seen between the two groups.

For all of the accuracy data, Mauchly’s test of sphericity was not significant. The Shapiro–Wilk normality test indicated that the accuracy data was normally distributed for both groups, and thus an ANOVA was performed. The behavioural data demonstrated that motor learning occurred as both the control (F(1,11) = 59.93, *p* < 0.001) and capsaicin (F(1,11) = 23.16, *p* < 0.001) groups showed decreases in error post-acquisition and following the retention test (from pre-test). The interaction effect of TIME by GROUP was significant (F(2,23) = 3.23, *p* < 0.05), with post-hoc ANOVA testing with a Bonferroni correction demonstrating that pre-test (F(1,11) = 18.88, *p* < 0.005), post-acquisition test (F(1,11) = 15.32, *p* < 0.005), and following the retention test (F(1,11) = 17.04, *p* < 0.005), the capsaicin group was more accurate than the control group (See Figure 4). Because the pre-test performance was different between groups, the performance data was normalized to each participant’s pre-test data before running the ANOVA. This confirmed that motor learning occurred as there was a significant effect of TIME (F(2,23) = 93.27, *p* < 0.005) with significant differences post-acquisition (F(1,11) = 61.21, *p* < 0.005) and at retention (F(1,11) = 192.81, *p* < 0.005) (see Figure 4). The effect of TIME by GROUP was not significant (*p* = 0.55), indicating that there was no difference in degree of improvement between groups when the baseline differences were taken into account. Relative to the pre-test values, the control group had a 48.7% decrease in mean motor error post motor acquisition and a subsequent 21.9% decrease at retention. The capsaicin group had a 35.2% decrease in mean motor error following motor acquisition and a subsequent 10.7% decrease at retention. The average time from the post-acquisition test to the retention test was 29.3 h (SD 5.6) for the control group and 34.5 h (SD 9.9) for the capsaicin group.

### 3.3. Transcranial IO Curves

**Figure 5:**
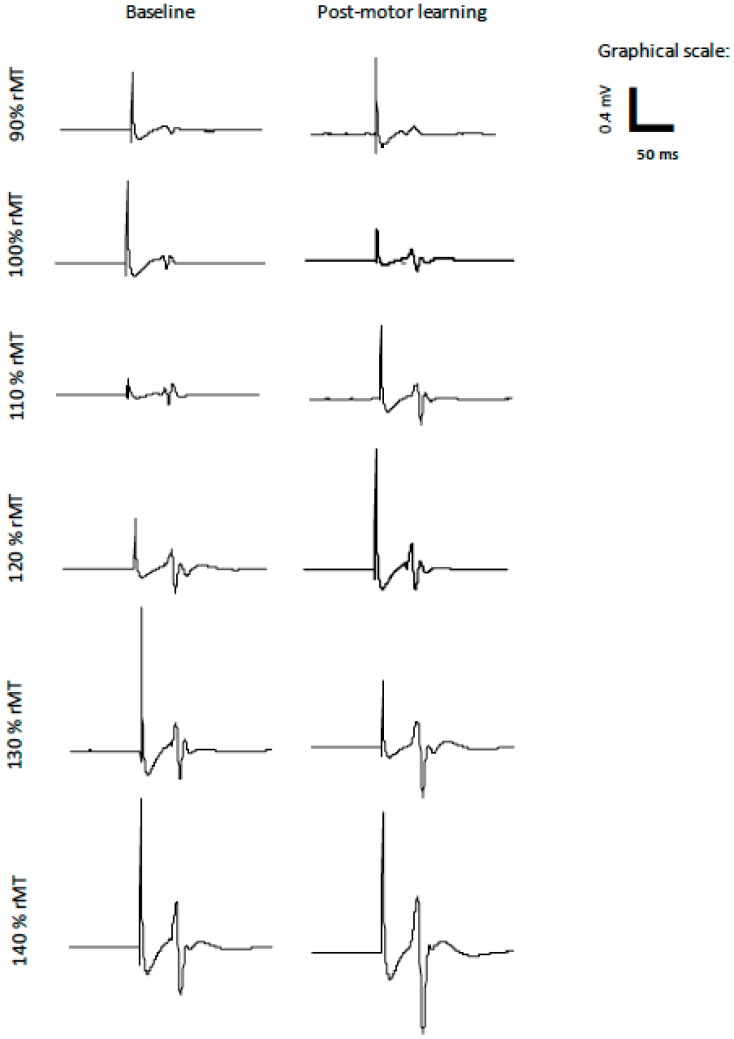
Average MEP data at baseline and post-motor learning from a representative control participant at all stimulation intensities (90% rMT–140% rMT).

**Figure 6:**
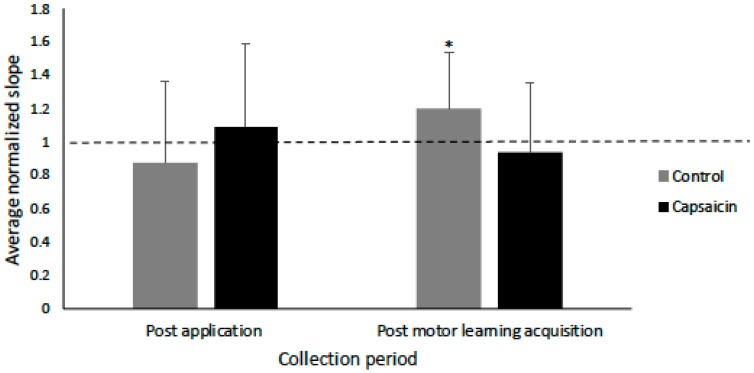
Bar-graph depicting the normalized average slope post-application and post-motor learning acquisition for the control and capsaicin groups. There was a significant increase in slope for the control group (*p* < 0.05) following motor learning acquisition as indicated by an asterisk. Error bars represent the standard deviation.

**Figure 7:**
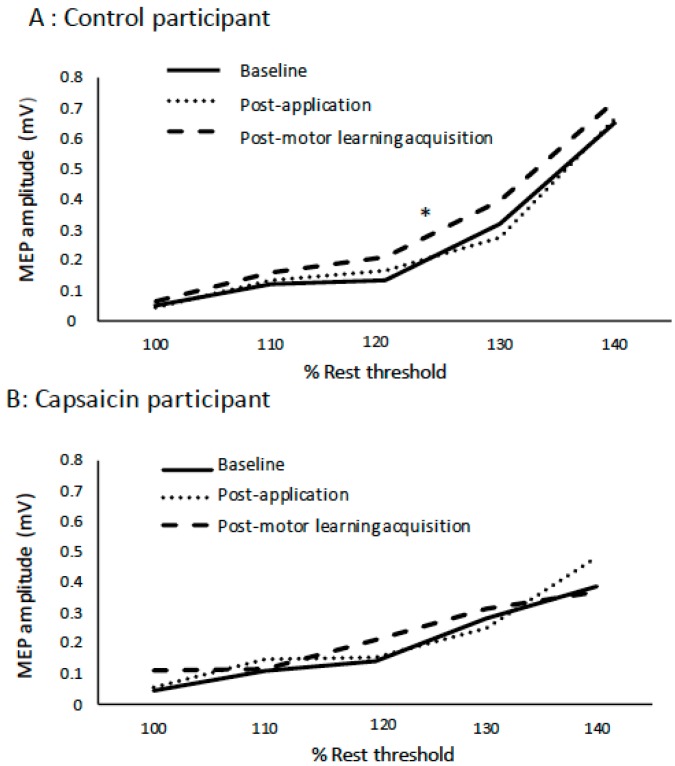
IO curves of control (**A**) and capsaicin (**B**) participants. Note increase in slope for the control (**A**) participants, representative of the significant change in the group data.

**Figure 8:**
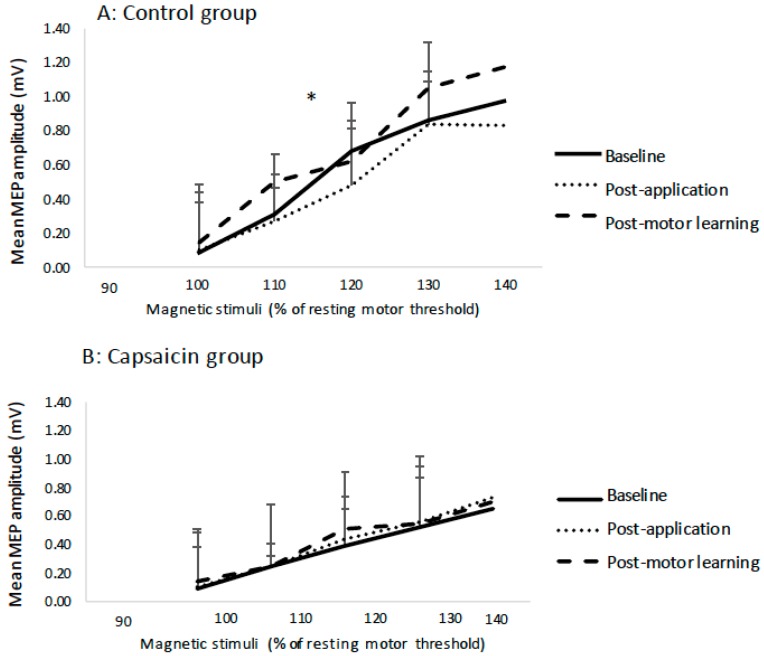
Mean IO curves of control (**A**) and capsaicin (**B**) groups. Note the increase in mean slope for the control (**A**) group as indicated by an asterisk. Error bars represent the standard deviation.

The slope data was normally distributed, and Mauchly’s test of sphericity was not significant. The average R^2^ value for the capsaicin group was 0.87 and for the control group was 0.84 (see Table 2). There was no effect of TIME on the IO slopes (*p* = 0.16) following cream application (capsaicin or placebo) or post motor acquisition. However, there was a significant TIME by GROUP interaction (F(2,23) = 3.69, *p* < 0.05), with post-hoc tests with a Bonferroni correction indicating that following motor acquisition there was a significant increase in slope for the control group (F(1,11) = 3.49, *p* < 0.05), and no significant change for the capsaicin group (*p* = 0.59). Figure 5 shows the average MEP data for a representative control participant at all of the stimulation intensities. The average IO slopes are illustrated in Figure 6. Figure 7 depicts example individual IO curves of typical control (A) and capsaicin (B) participants and Figure 8 depicts the mean IO curves for control (A) and capsaicin (B) participants.

## 4. Discussion

Our findings support our hypothesis that a motor task performed in a pain-free condition (controls) as compared to acute tonic pain (capsaicin group) would show an increase in the slope of the TMS IO curves.

Improvements in accuracy were found for both groups (during post-acquisition and retention tests), demonstrating that motor learning had occurred. The normalized post-acquisition/retention data demonstrated that both groups showed similar improvement in accuracy following acquisition and at retention. Pain is likely to have impacted motor performance throughout the study as the capsaicin group outperformed the control group pre-test, post-acquisition test, and following the retention test, corroborating our previous research [3,4]. Previous research demonstrated improved early motor learning with acute tonic pain, and it was hypothesized that this was due to increased attention to the limb performing the task as improvements in performance were seen with remote pain (applied to the same limb) as compared to a control condition [3,4] and with local pain as compared to remote pain [4]. Other work has demonstrated that acute experimental pain did not negatively impact acquisition and retention [15]. The acute tonic pain stimulus (capsaicin) used in the current study and our previous work [3,4] is similar to that used in the Bilodeau study [15]. This tonic cutaneous pain was unrelated to the motor task, and thus it is hypothesized that pain may only have a negative impact on motor performance when it directly impacts the ability to perform a motor task.

Our results demonstrated an increase in the slope of the mean IO curve for the control group following motor learning which is in line with the theory that there is an increase in excitability in the M1 that complements motor learning. However, there was no change in the mean slope of the IO for the capsaicin group. This suggests that, although an increase in the excitability of the M1 often accompanies improved motor performance, it is not essential for improvements to occur as both groups demonstrated motor learning acquisition and retention. These results can also be interpreted from a cognitive perspective. Increased excitability of the M1 accompanies cognitive decline in Alzheimer’s disease, and this may be compensatory following the loss of neurons that occurs with this disease [52]. Our current findings substantiate our previous work [42] in which we found differential SEP peak amplitudes (N18) for participants who performed motor acquisition with capsaicin applied to the elbow similar to this study (remote pain, as compared to contralateral and local pain groups), but with no differences in performance accuracy between groups. These results are in line with Cirillo [53], who also found that there was no association between MEP changes and improved motor performance. Other work has found that increased MEPs are associated with motor learning, and this correlates with improved performance [8,54]. Increased IO slope excitability reflects the initial early (fast) motor learning stage [54,55]. There is an increase in MEPs following motor performance of the Purdue pegboard [56] or finger [8] tasks. It has also been revealed that this increase in MEPs following motor learning may be transient [9]. There was an increase in MEPs following a pinch task with a return to baseline once learning saturation occurred [39] and an increase in the cortical maps of the muscles involved in a serial reaction time test with a return to baseline values once the task was explicitly learned [9]. The fast motor learning stage occurs when there is a within-session improvement and is induced by a few trials on a time scale of minutes [40]. The increase in excitability of the M1 is associated with early stage (fast) motor learning and is not present once a skill has been learned. This may provide an explanation as to why there was not a significant increase in the slope post-acquisition test for the capsaicin group as this group was more accurate initially.

The TMS testing could be performed at baseline, post capsaicin, and post motor acquisition. However, all performance testing for the capsaicin group, including the baseline pre-acquisition test, was performed in the presence of pain. As described in the introduction, if we had performed the baseline testing without capsaicin and then performed the post-acquisition test in the presence of capsaicin, we would not have known if the performance improvements reflected motor learning or the effects of the capsaicin. Hence, as described in the introduction, we intentionally chose to perform the pre-acquisition testing in the presence of pain in order to uncouple motor learning effects on performance from pain effects on performance. This means that we cannot definitively say that the improved pre-acquisition performance was due to capsaicin. However, given that two past studies [41,42] showed that capsaicin led to better motor performance, it is likely that the improved baseline was due to the presence of capsaicin.

The IO curve is affected by the excitability and distribution of excitability of interneurons and motoneurons at cortical and spinal locations [57]. Therefore, it could be argued that the alterations in excitability following motor learning are due to peripheral or spinal mechanisms as opposed to alterations at the level of the M1. However, a prior motor learning study utilized TMS and TES (transcranial electrical stimulation) and found that there was an increase in excitability following TMS but not following TES. TES activates the corticospinal tract directly while TMS activates this tract transynaptically, and therefore it was suggested that the alterations in excitability following motor learning are occurring primarily at the level of the cortex [58]. However, there is also adaptive plasticity at the spinal level that occurs with motor learning [59] which may occur through presynaptic inhibition at the spinal level learning [60], and therefore we cannot say definitively whether the alterations in excitability are occurring at the cortical, subcortical, or spinal level.

Currently, the mechanism responsible for changes in CSE are not fully understood. It is known that the increases in MEPs associated with motor performance are impacted by GABAergic intracortical inhibition [54], and it is hypothesized that disinhibition in the M1 plays an important role in the early stages of motor learning [61,62,63]. In addition, synaptic neuroplasticity in the M1 is proposed to be facilitated by the activation of the N-methyl-D-aspartate (NMDA) receptor [64]. In addition, a decrease in GABA inhibition and an increase in glutamate provides a mechanism for the increase in M1 excitability following motor learning for the control group. A reduction in GABA-mediated mechanisms of inhibition has been observed in different clinical scenarios, corroborating that a “disinhinibited” M1 plays an important role in the early stages of motor learning [65,66,67]. In addition, the synaptic neuroplasticity in the M1 is most likely facilitated by the activation of the NMDA receptor [68,69,70]. In addition, animal studies have suggested that long term potentiation (LTP) and long term depression (LTD) of synapses contributes to the neuroplasticity associated with motor learning [62,71] and provides a mechanism for increased excitability following motor learning.

A recent study by Mavromatis, Neige [44] examined the interactive effect of capsaicin cream and a pinch motor task on corticospinal excitability as measured by TMS (using SICI). Similar to our findings, this study found that the control group had an increase in excitability that was not observed for the capsaicin group and did not find a negative impact of pain on motor performance outcomes. Our current finding corroborates previous work that found differential effects of pain and early motor learning on neuroplasticity as measured by SEPs when compared to a control group, without a negative impact on motor performance [3,4,41].

## 5. Conclusions

The current study suggests that there may be contradictory effects of acute pain on motor learning neuroplasticity. Research indicates that cortical representations in the primary somatosensory area (S1) and M1 are altered in response to pain, and that pain perception and the associated alterations in neuroplasticity can be reversed by motor learning [72]. Clearly there is a link between motor and sensory systems and the effects of pain on motor learning may be due to cortico-thalamic, cortico-cerebellar, or cortico-cortico loops.

## 6. Limitations

Given that capsaicin causes vasodilation that is mediated locally [73], it is possible that some of the effects of MEPs that were observed in response to acute experimental pain may have been due to other local effects (i.e., skin temperature) as opposed to a direct effect of pain on MEP peak amplitude. Another limitation of this study is that we could only collect one set of pre-acquisition data due to learning effects, and therefore we did not collect pre-motor learning accuracy data before the capsaicin was applied, so it is possible that the two groups differed in performance at baseline, unrelated to the capsaicin application. However, as mentioned, two previous studies using the same task [41,42] found that the presence of remote capsaicin-induced pain improved initial motor performance, making it likely that the baseline differences were indeed due to capsaicin. There were some limitations to our study design as we used resting as opposed to active TMS IO curves to assess adaptation of a motor task. In addition, we did not measure the compound motor action potential (CMAP). As the ratio between the maximal (transcranially evoked) MEP amplitude and CMAP is believed to reflect the central mechanisms contributing to the MEP amplitude, we would have minimized inter-individual differences, and we could make stronger conclusions about the effect of pain and early motor learning on M1 excitability.

This study found an increase in slope for a control group following an acquisition phase that was not observed for the capsaicin group. A future direction for a study would involve the measurement of cortisol levels in response to acute cutaneous pain and early motor learning as endogenous stress hormones are a component of a memory modulating system that results in memory strength proportional to memory importance [74]. In addition, performing TMS IO curves midway through the acquisition phase could demonstrate whether there is an increase in slope for a capsaicin group at this time point that has already returned to baseline by the time measured in the current study. In addition, another future study could include the application of the cream to remote locations (contralateral arm or the leg) and the evaluation of CSE from both hemispheres, in order to obtain bilateral data to compare and to speculate on mechanisms of transcallosal interaction (and possibly inhibition).

## Figures and Tables

**Table 1:** Resting motor threshold (rMT) values that were determined by finding the lowest stimulator intensity that elicited an MEP of at least 0.05 mV in at least five out of ten trials.

Participant	Control	Capsaicin
1	36	62
2	49	54
3	58	60
4	62	49
5	44	52
6	64	53
7	45	40
8	53	42
9	43	56
10	59	64
11	57	47
12	42	53
Averages	51.82	52.67
SD	9.02	7.40

**Table 2:** Goodness of fit data (R^2^) for the capsaicin and control groups at baseline, post-application, and post-motor learning acquisition.

	Control			Capsaicin		
Participant	Baseline	Post-Application	Post-Motor Learning	Baseline	Post-Application	Post-Motor Learning
1	0.90	0.80	0.95	0.86	0.77	0.75
2	0.88	0.88	0.91	0.74	0.96	0.95
3	0.77	0.76	0.87	0.78	0.85	0.89
4	0.78	0.70	0.90	0.92	0.75	0.92
5	0.93	0.89	0.87	0.87	0.78	0.84
6	0.90	0.80	0.95	0.96	0.93	0.89
7	0.67	0.78	0.74	0.88	0.81	0.88
8	0.90	0.86	0.90	0.93	0.95	0.76
9	0.84	0.83	0.72	0.92	0.87	0.79
10	0.85	0.97	0.97	0.87	0.90	0.96
11	0.82	0.94	0.91	0.88	0.85	0.89
12	0.82	0.88	0,80	0.96	0.93	0.88
Averages	0.84	0.84	0.88	0.88	0.86	0.87
SD	0.073	0.077	0.081	0.067	0.073	0.069
